# Virtual social interactions during the COVID-19 pandemic: the effect of interpersonal motor synchrony on social interactions in the virtual space

**DOI:** 10.1038/s41598-023-37218-6

**Published:** 2023-06-28

**Authors:** Hila Gvirts, Lya Ehrenfeld, Mini Sharma, Moran Mizrahi

**Affiliations:** Department of Psychology, Ariel University, Ariel, 9851328 Israel

**Keywords:** Cognitive neuroscience, Social behaviour, Social neuroscience

## Abstract

Although the link between motor synchrony and emotional alignment has been extensively studied during face-to-face interaction, the question of whether this association also exists in virtual settings has remained unanswered. Here, we examined whether this link exists during virtual social interactions and whether pro-social effects will be induced during those interactions. To this end, two strangers shared difficulties they have experienced due to the COVID-19 pandemic during a virtual social interaction that included both audio and video. The findings revealed that motor synchrony and emotional alignment can arise spontaneously during a virtual social interaction between two strangers. Moreover, this interaction yielded a decrease in negative affect and an increase in positive affect, as well as an increase in feelings of trust, liking, cohesion, self-other overlap, and similarity between the strangers. Finally, a higher level of synchrony during the virtual interaction was specifically associated with increased positive emotional alignment and liking. It can thus be presumed that virtual social interactions may share similar characteristics and social effects as face-to-face interactions. Considering the tremendous changes the COVID-19 pandemic has caused regarding social communication, these findings may provide grounds for developing new intervention protocols aimed at dealing with the consequences of social distancing.

## Introduction

### The role of motor synchrony during social interactions

Motor synchrony serves as an integral part of human social behavior^1^, and it can emerge both intentionally and spontaneously. Intentional motor synchrony occurs when two or more people purposely synchronize their motor movements. For example, dancers align their movements purposefully to synchronize with one another^2^. Alternatively, spontaneous synchrony occurs when two or more people synchronize their body movements during daily interactions unintentionally and without awareness^3^. For example, walking steps and pace may spontaneously synchronize without awareness while taking a walk together with another person^4^. The mere visual exposure between two people may unconsciously lead to synchronized body postures^5^. Similarly, it is normally difficult not to smile back at a person smiling at us^6^.

Synchrony is a basic process in human communication that takes place immediately after birth within mother–child interactions^7^. It has a crucial role in the process of forming interpersonal relationships^8,9^ and serves as an integral part of our social behavior and cognition^1,10^. Specifically, motor synchrony facilitates pro-social behavior^11,12^ as well as enhances liking^13–15^, trust^16^, and cohesion^13,17,18^ between individuals interacting face-to-face. Likewise, participants singing together in synchrony were found to report higher levels of similarity to the group than participants who did not sing in synchrony^19^. However, some studies did not replicate this association between motor synchrony and pro-social effects^20,21^. For example, interacting with a virtual avatar programmed to mimic participants’ movement, did not elicit greater feelings of trust, similarity and self-other overlap compared to interacting with an avatar that was not programmed to mimic movement^21^.

Similar to the tendency to synchronize movements to one another during social interactions, people also tend to catch the emotions of others around them^22^. This tendency can occur both consciously or unconsciously and is referred to as emotional contagion (i.e., emotional alignment). The connection between motor synchrony and emotional alignment can be attributed to a shared neural mechanism that involves a feedback loop comprising of three components.: (a) an error-monitoring system that reacts to misalignment, (b) an alignment system, and (c) a reward system that is activated when alignment is achieved^23^. Notably, it has been suggested that the activation of either motor synchrony or emotional alignment elicits the activation of the other by activating the feedback loop^23^. Supporting evidence has demonstrated that inhibition of motor synchrony between participants interferes with emotional alignment while listening to emotional vocalizations^24^. Moreover, motor synchrony and emotional alignment were found to adhere to similar principles. For instance, group membership affects both motor synchrony and emotional alignment; that is, people tend to synchronize^25^ and catch the emotions^26^ of ingroup rather than outgroup members. Additionally, both synchrony and emotional alignment were found to enhance feelings of closeness^27–29^.While this line of evidence supports the link between synchrony and emotional alignment during face-to-face interaction and their association with pro-social effects, the question of whether these associations also exist in virtual settings has remained unanswered.

Motor synchrony and emotional alignment are two phenomena that are mediated by the mirror neuron system^23^ or the action-observation network^30^. This network enables individuals to anticipate the intentions of others by observing their actions. As a result, the same brain regions are activated when we perform the same movements and experience the same emotions, allowing the sharing of bodily states. Recent neuroscience studies support the idea that emotions are embodied and that bodily sensations play a significant role in emotional experiences^31^. Embodied simulations enable us to better understand others and align our behavior and emotions with our environment, which is essential for the development of empathy^32–35^. Embodied simulations may also blur the boundaries between oneself and others, increase perceived similarity^32^, encourage liking^32,36^, cohesion^32^, and trust^37^.

Modern technology has changed the way we socialize by incorporating virtual social interactions, such as text messaging, audio, and video calls, into our daily life^38^. The outbreak of the COVID-19 virus has further changed the way people communicate with one another by forcing them to rely much more on technology for communication than ever before. With worldwide restrictions on face-to-face interactions, virtual social interactions provided a substitute^39^. Previous studies have shown that although virtual interactions can be very beneficial, they still may reduce interactional engagement^40^ and have a cognitive cost for creative idea development collaboration capacity^41^. Thus, the COVID-19 era has stressed the need to investigate the nature of virtual social interaction; more specifically, whether core phenomena such as the human tendency to spontaneously synchronize body movements and align emotions exists in virtual settings and whether pro-social effects arise in these settings. In other words, could virtual social interactions satisfy our need for social connection and mitigate loneliness?

### The present research

In the present research, we examined whether virtual social interactions that include both audio and video provide a similar opportunity for connectedness as face-to-face social interactions by using the Zoom software. To examine this question, we asked two strangers to share difficulties they have experienced due to the COVID-19 pandemic during a virtual social interaction. These instructions were aimed at motivating participants to share emotional experiences. We expected that this shared experience would elicit both emotional alignment and spontaneous motor synchrony between the two strangers while they interacted with each other.

We used Motion Energy Analysis (MEA) – a computer-based tool that automatically and objectively quantifies motor synchrony during natural interactions^42^– to measure the level of interpersonal synchrony during the interaction. The MEA tool allows for measuring dynamic movements between interacting partners under natural conditions^6^, thus enabling subtle examination of synchrony, such as the level of synchrony in therapist-patient dyads during therapy sessions^43^. To measure emotional alignment, we used the Positive and Negative Affect Schedule (PANAS)^44^ which lets participants report their positive and negative affect at the current moment. The ability to assess affect at the moment enables detecting the change in the emotional state following the interaction. Further explanation of the specific calculations made to examine emotional alignment are in the results section. All pro-social effects were examined using self-report measures which will be described in greater detail in the measures section.

Our first goal was to validate the new task; that is, to demonstrate that emotional alignment and motor synchrony may arise spontaneously during virtual social interactions. Furthermore, as mentioned earlier, face-to-face interactions were previously found to promote pro-social effects such as trust, liking, cohesion, self-other overlap, and perceived similarity^12–18^. Here, we examined whether the same pro-social effects that have been found during face-to-face interactions will also be replicated in virtual settings.

## Method

### Participants

One-hundred and ninety-six male and female Hebrew-speaking students between 19 and 30 years old were recruited for the study. All were students at Ariel University, who received credits that are required for their program. Registration for the experiment was made through a computerized system at Ariel University**.** All methods were carried out after obtaining the necessary approvals from the Institutional Review Board at Ariel University. Additionally, all methods were carried out in accordance with relevant guidelines and regulations. Informed consent was obtained from all the participants in the study. Participants were randomly assigned into ninety-eight dyads, while ensuring all dyads were made up of two same-sex strangers without previous encounters. Each dyad conducted a virtual social interaction via the Zoom application using their personal computers and webcams. All participants were encouraged to sit in a calm, distraction-free environment with a stable internet connection. Twenty-nine dyads were excluded due to technical issues such as unstable internet connection or lag in audio and video that did not allow proper MEA analysis (see full description of MEA prerequisites: Ramseyer^45^). The final sample included 138 participants (22 males and 116 females) assigned in 69 dyads.

### Measures

#### Motion energy analysis

**Figure 1:**
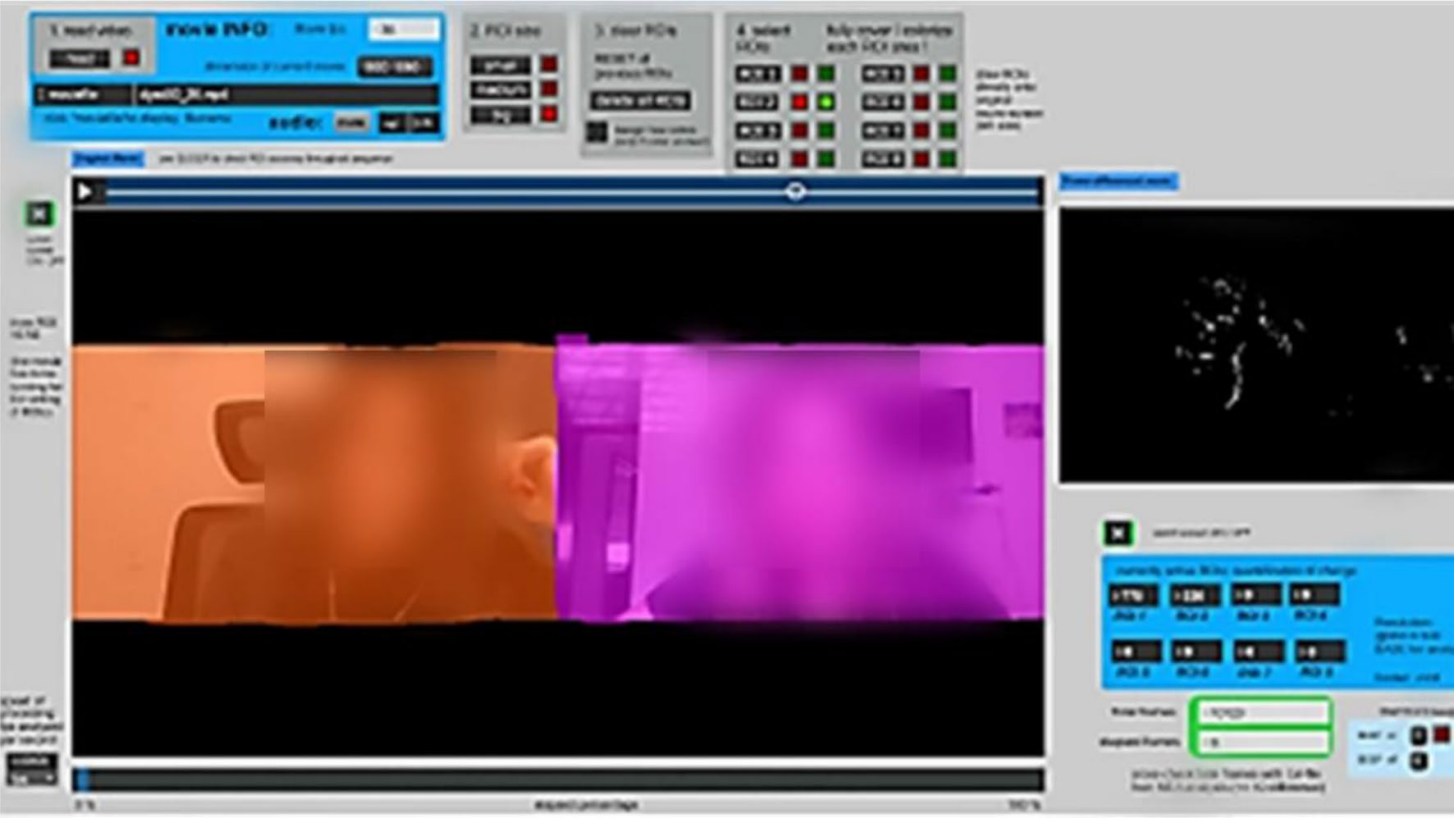
A snapshot of the MEA software, while analyzing one video of our collected data. The two predefined regions of interest (ROI) appears in two different colors, each capturing the head and shoulder area of a single participant.

The MEA is an automated method to calculate motion synchrony between individuals. This software allows us to convert pixels from digital videos of the interaction into their greyscale format. Each frame has a fixed number of pixels that represent the distribution of grey-scale values ranging from 0 (true black) to 255 (true white)^46^. MEA allows to draw the region of interest manually and, therefore, tracks the change in “energy”, which is defined as changes from one video frame to the next and stores the amount of change occurring in a defined ROI into a time‐series representative of the movement that occurred in this ROI^47^(see Fig. 1 for an illustration of the software). These time-related changes are indicators of movement within specific ROIs. The absolute changes in greyscale values in these ROIs will be detected and separately recorded, thus generating two continuous time series measuring the amount of total full-body movement of each participant (for full description see Ramseyer, 2020^45^).

To quantify automatically coded synchrony, we used a readily accessible and well-documented program designed to conduct motion energy analysis (rMEA in the R package, for details, see Ramseyer & Kleinbub^48^). The primary statistical analysis was based on a time‐lagged cross‐correlation algorithm (function MEAccf in rMEA, see Boker et al.^49^). Briefly, cross-lagged correlations were applied to quantify the synchrony of the preprocessed motion energy time series of the participants in each dyad. Correlations between the time series of each dyad were computed such that a lag zero correlation reflects simultaneous synchrony. We divided the 5-min time series into 60 s segments overlapping by 30 s with a time lag window of 5 s (i.e., ± 5 s in both directions). Cross correlations were then standardized (Fisher’s Z) and their absolute values aggregated for every 60 s segment, yielding one global value of simultaneous synchrony. These parameters were chosen according to the guidelines suggested by Ramseyer^45^ and the nature of our data. Note that the use of absolute values, as suggested by Ramseyer and Tschacher^43^, means that both positive and negative cross-correlations contributed positively to synchrony.

In order to control for the possibility that synchrony occurred by coincidence, we ran a pseudo-synchrony analysis (i.e., compared the real associations found in genuine dyads with chance associations produced by pseudo dyads). To create the pseudo interactions, we shuffled the original time series of each dyad, then aligned movement segments of different interaction partners that never actually occurred at the same time (i.e., 9660 pseudo dyads were created by pairing a participant from one dyad with a participant from another dyad). To calculate the level of pseudo dyadic synchrony, we used the same analysis as for real dyads, which yielded one global value of simultaneous synchrony for each pseudo dyad.

#### Self-report measures

##### Interpersonal trust

The trust questionnaire is a 5-item measure developed by Murray et al.^50^ based on a measure developed by Holmes et al.^51^ It measures the degree of trust in the partner’s continuing motivation to be responsive to one’s needs on a Likert scale ranging from 1 (strongly disagree) to 5 (strongly agree) (e.g., “When we are dealing with an issue that is important to me, I feel confident that my partner will put my feelings first,” “I feel that I can trust my partner completely”). The internal consistency in our data was adequate before the interaction (α = 0.72) and following the interaction (α = 0.76).

##### Liking

The liking questionnaire is a 4-item measure developed by Mackinnon et al.^52^. It measures the degree of liking towards another person on a Likert scale ranging from 1 (strongly disagree) to 5 (strongly agree) (e.g., “How friendly do you think the person would be if you met this person in real life?”). The internal consistency in our data was adequate before the interaction (α = 0.80) and following the interaction (α = 0.86).

##### Cohesion

The cohesion scale is a 5-item questionnaire previously used by Wiltermuth and Heath^19^ and Cross et al.^53^ It measures trust, mood, and cohesion (closeness, connectedness, and similarity). Since some of the items overlap with other questionnaires we used, we only included 3 items ("How similar do you feel to the other participant?", "How connected do you feel to the other participant?", "How much do you trust the other participant?"). Participants recorded their responses to each of these questions by marking the continuum. This response scale was used to make it more likely to detect any changes after the movement manipulation and has been successfully used in a similar context by Lumsden et al.^9^ The internal consistency in our data was adequate before the interaction (α = 0.87) and following the interaction (α = 0.93).

##### Self-other overlap

Self-other overlap was measured using the Inclusion of Other in Self (IOS), a single item, pictorially measure developed by Aron et al.^54^ It is intended to tap directly into people's sense of interpersonal interconnectedness in a nonverbal way. In the IOS scale, respondents select the picture that best describes their relationship from a set of Venn-like diagrams, each representing different degrees of overlap between two circles. The figures were designed so that (a) the total area of each figure is constant (thus as the overlap of the circles increases, so does the diameter), and (b) the degree of overlap progresses linearly, creating a seven-step, interval-level scale. The IOS is a simple, well-validated, and commonly used scale.

##### Positive and negative affect

The Positive and Negative Affect Schedule (PANAS) is a 20-item measure developed by Watson et al.^44^ It is a reliable and valid tool for measuring the two different mood states (positive and negative affect) over different intervals of time (‘today’, ‘during the past few days’, ‘during the past year’, ‘in general on an average’, etc.). The internal consistency in our data was adequate for the PA subscale before the interaction (α = 0.87) and following the interaction (α = 0.86), as well as for the NA subscale before the interaction (α = 0.83) and following the interaction (α = 0.84).

##### Perceived similarity

The perceived similarity was measured using a perceived similarity questionnaire^55^ which includes 25 items that are rated on a Likert scale ranging from 1 (strongly disagree) to 5 (strongly agree). The questionnaire is divided into two sub-scales—one examines similar backgrounds and includes 10 items, and the second examines similar attitudes and includes 15 items. The internal consistency in our data was adequate before the interaction (α = 0.89) and following the interaction (α = 0.89).

### Procedure

**Figure 2:**
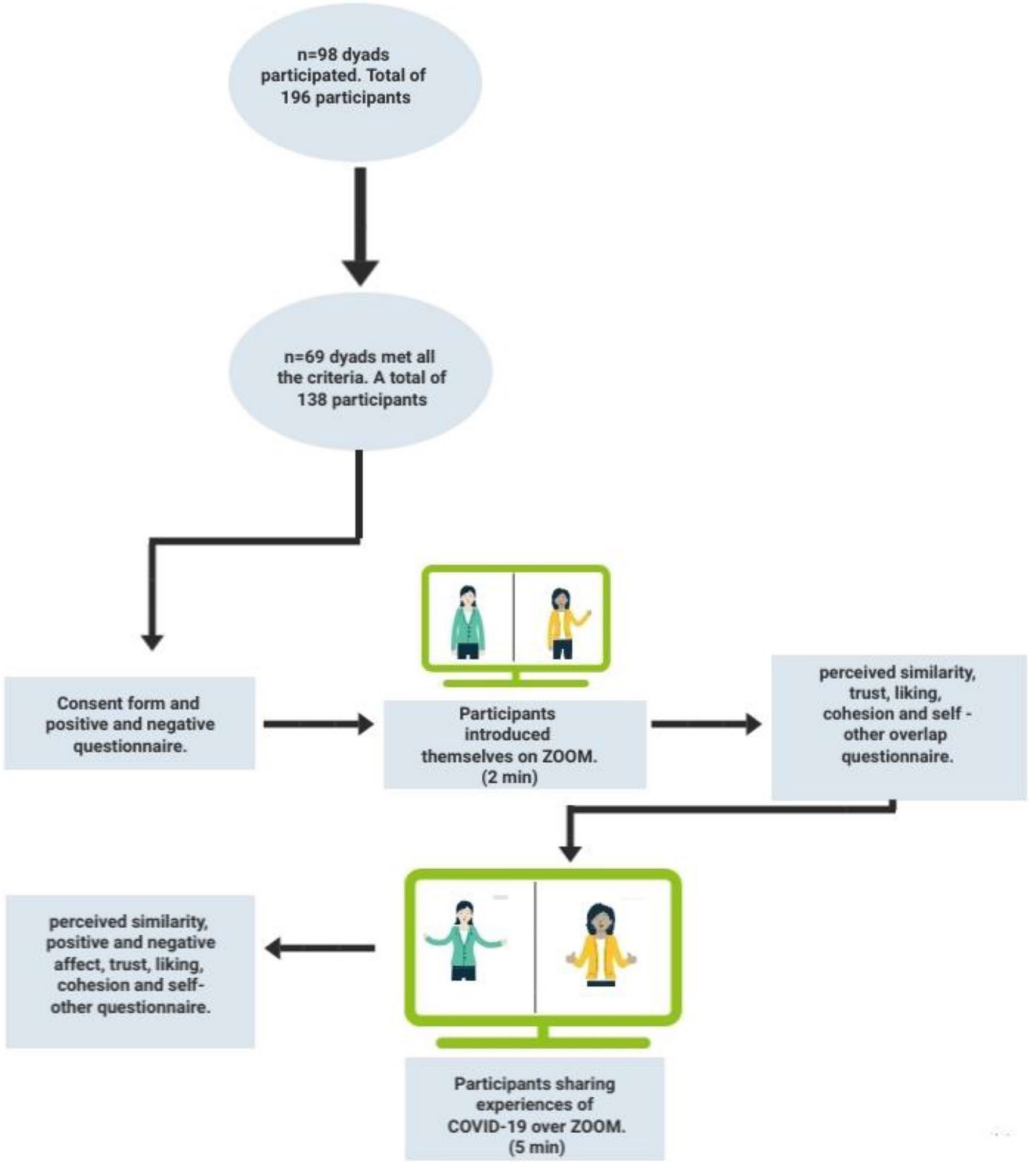
Experimental flowchart.

As shown in Fig. 2, before the beginning of each session, each participant received an e-mail including informed consent, the subject's number, the dyad's number, a link to the Zoom meeting, and lastly, a questionnaire investigating demographic information and the positive and negative affect schedule questionnaire (PANAS), which had to be filled out before entering the Zoom meeting. In each Zoom meeting, one dyad and one research assistant were present. Upon joining the Zoom meeting, the research assistant instructed participants to use the pin option to view their interaction partner on full screen and to give a short introduction of themselves to one another for the next two minutes. At the start of the experiment, the research assistant turned off their camera and microphone. After two minutes, they turned them back on and instructed the participants to turn off their own cameras and microphones. The purpose was to allow the participants to fill out a questionnaire measuring trust, liking, perceived similarity, self-other overlap, and cohesion. Next, the research assistant instructed participants to turn on cameras and microphones and instructed them to share the difficulties they are experiencing with COVID-19 and how it affected their personal lives for the next five minutes (see Fig. 2). It is worth noting that the experiment took place between April 25^th^ and May 9^th^, 2021, a time in which many social distancing and quarantine restrictions were still enforced, such as remote learning at universities. The research assistant turned off the camera and microphone during the dyadic conversation. After five minutes, the research assistant returned the camera and microphone and asked participants to finish the talk and switch off the cameras and microphones while filling out the last questionnaire measuring trust, liking, perceived similarity, self-other overlap, cohesion, and positive and negative affect (see Fig. 2). To further analyze the films, we only chose those that met the MEA's basic criteria, such as a static background in the video, stable lighting, and a camera^45^. Due to the background noise in some videos, a few frames were manually eliminated. The resolution of all recorded interactions was standardized to 1920 × 1080 pixels at 30 frames per second. The virtual interaction was recorded via Zoom and transferred and saved to a private cloud. The video recordings were deleted after all analysis were completed.

## Results

### Task validation

#### Virtual interaction induces motor synchrony

**Figure 3:**
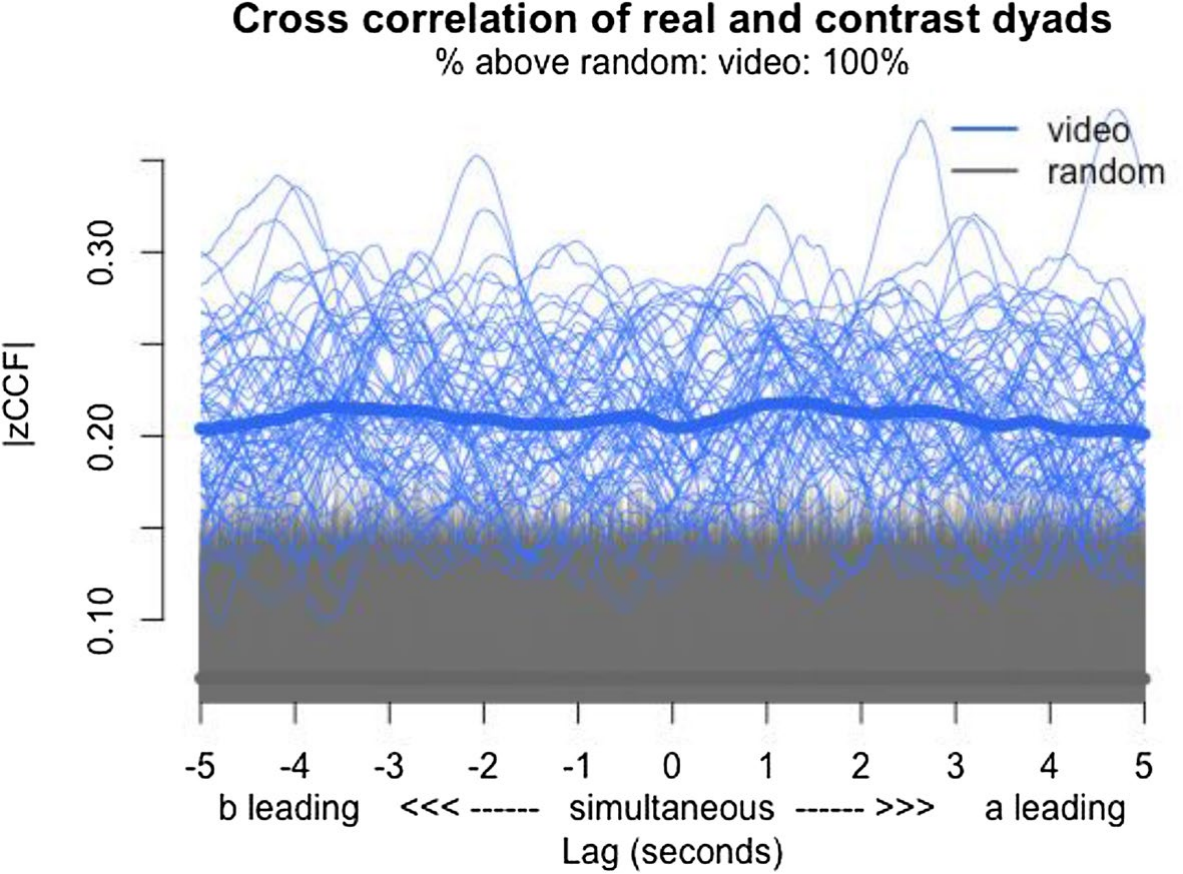
The lag-plot comparing real dyads and pseudo dyads (colored lines: real = blue and pseudo = grey line): Y axis = absolute cross-correlation (= synchrony), X axis = lags.

**Figure 4:**
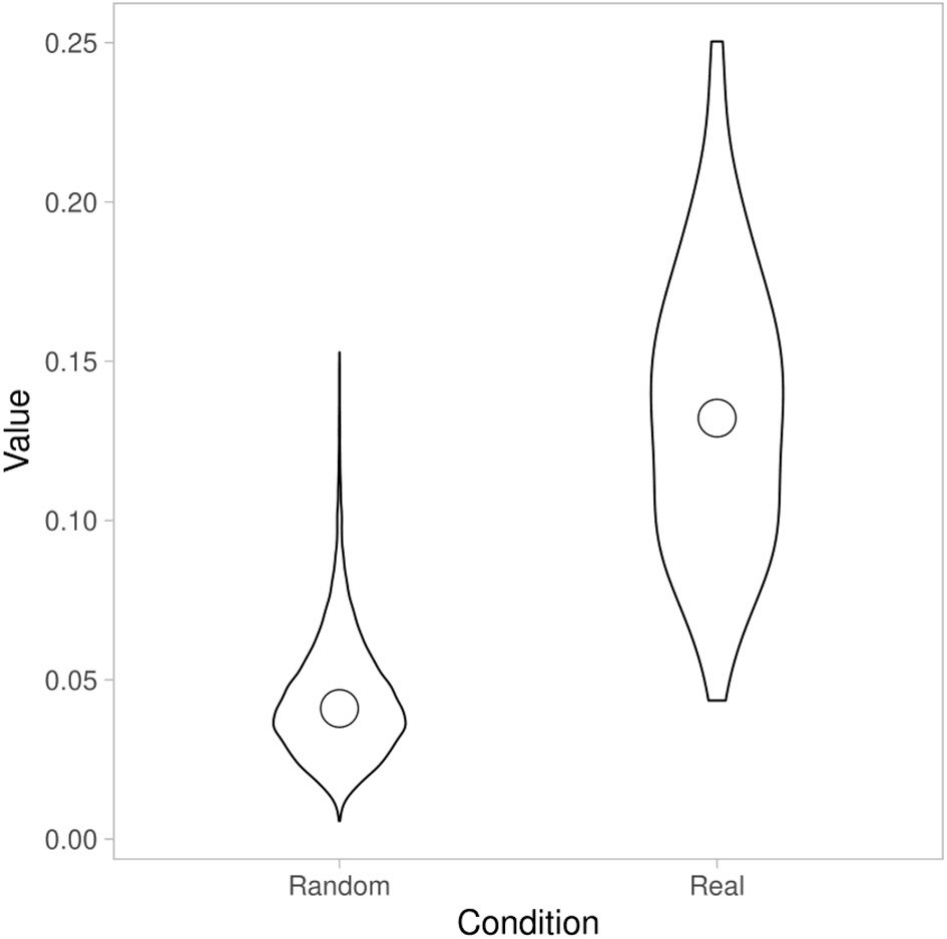
Shows a violin chart of the distribution of all dyadic synchronization levels within our data. On the right, we present the real data set (69 dyads) and on the left all pseduo-dyads (9660 shuffled dyads). As can be seen, the real data set comprises higher synchrony compared to the shuffled data, supporting the notion that synchrony as measured by the MEA emerged during virtual interaction. Spontaneous synchrony was significantly higher in real dyads than in pseudo-dyads, suggesting that synchrony is due to the dyadic interaction and not spurious.

To validate our task, that indeed motor synchrony occurred during the virtual social interactions, we employed a two-tailed independent-samples t-test, with group (real dyads vs. pseudo dyads) as the independent variable and global value of simultaneous synchrony as the dependent variable. Results showed that synchrony in real dyads (*M* = 0.14, *SD* = 0.04) was significantly higher than synchrony in pseudo dyads (*M* = 0.04, *SD* = 0.02), (*t* (9727) = 39.92, *p* = 0.000), suggesting that participants spontaneously synchronized movement with each other during the virtual social interaction (see Fig. 3 and 4).

#### Virtual social interaction induces emotional alignment

In order to determine whether participants experienced emotional alignment, we calculated the difference in negative emotions by extracting the absolute values of the two dyad members’ negative affect scores (i.e., the difference in negative affect index) using the NA subscale of the PANAS questionnaire. A similar index was calculated for the positive affect scores (i.e., difference in positive affect index) using the PA subscale of the PANAS questionnaire. Note that a lower difference score in these indexes reflects higher similarity in emotional state (i.e., emotional alignment).

**Figure 5:**
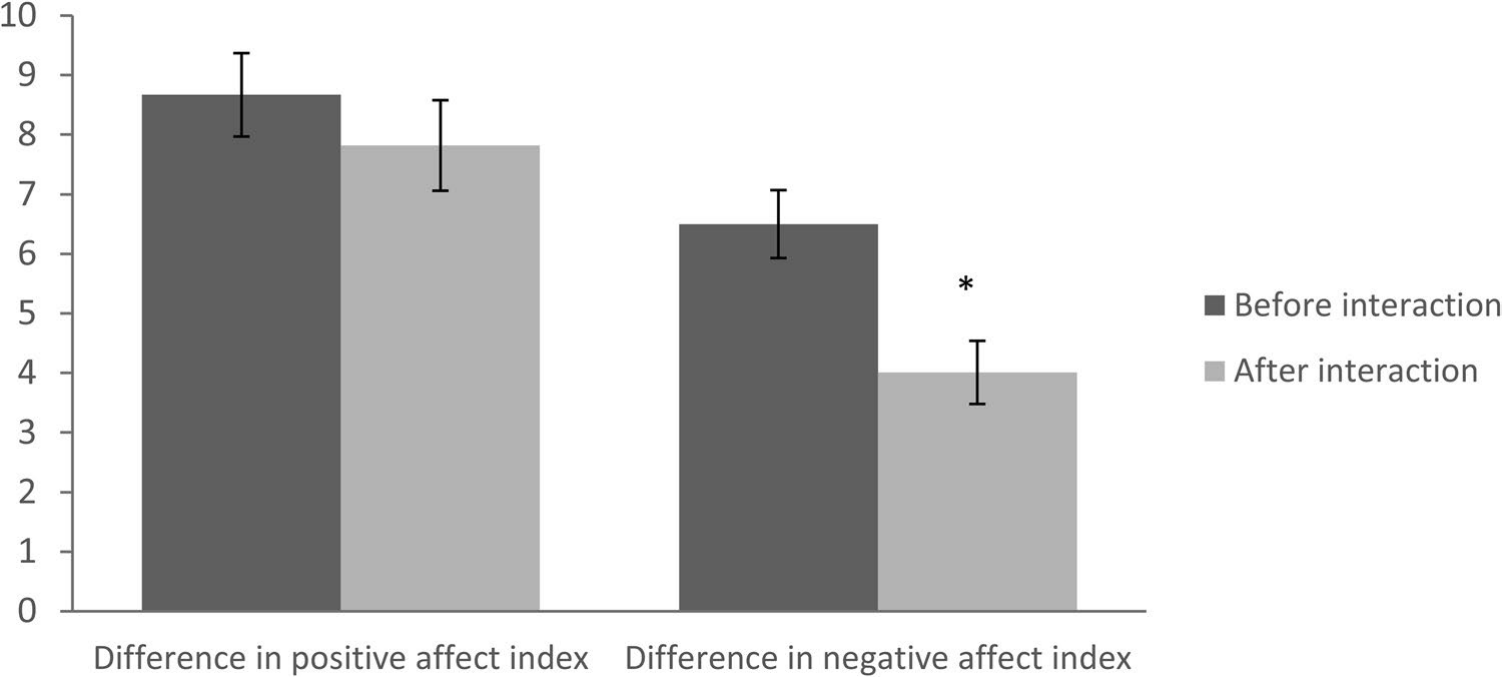
On the left side of the figure, the difference in positive affect index before and after the interaction is displayed, while on the right side, the difference in negative affect index before and after the interaction is shown. The composite scores were computed based on the difference between the participant's self-report and their interaction partner's report. Separate composite scores were calculated for negative affect and positive affect before and after the interaction. It is important to note that a lower score indicates a higher level of emotional alignment.

Next, to validate our task, that indeed emotional alignment occurred during the virtual social interactions, we employed two two-tailed paired-sample t-test. One test with time (before interaction vs. after interaction) as the independent variable and difference in positive affect as the dependent variable. A second test was used with time (before interaction vs. after interaction) as the independent variable and difference in negative affect as the dependent variable. The difference in the negative affect index before the interaction (*M* = 6.50, *SD* = 4.74) was significantly higher than the difference in the negative affect index after the interaction (*M* = 4.01, *SD* = 4.39); *t* (68) = 4.12, *p* = 0.000 (see Fig. 5). However, the difference in the positive affect index before the interaction (*M* = 8.67, *SD* = 5.80) was not significantly higher than the difference in the positive affect index after the interaction (*M* = 7.83, *SD* = 6.35); *t* (68) = 1.12, *p* = 0.268 (see Fig. 5). Thus, suggesting that the virtual interaction elicited emotional alignment between participants in negative affect.

#### Synchrony's pro-social effects

To examine whether a social virtual interaction that includes both audio and video induces pro-social effects, we conducted repeated measures multivariate analysis of variance (MANOVA) with time (before and after the interaction) as a within-subjects factor. This analysis allowed us to determine whether a significant difference exists between the average pro-social scores (i.e., dyad average score on positive and negative affect, trust, liking, cohesion, self-other overlap, and perceived similarity) before and after the interaction.

Within-subjects MANOVA results showed a significant difference between the average dyad score on positive affect (*F* (1, 68) = 32.82, *p* < 0.000), negative affect (*F* (1, 68) = 27.03, *p* = 0.000), trust (*F* (1, 68) = 45.25, *p* = 0.000), liking (*F* (1, 68) = 40.02, *p* = 0.000), cohesion (*F* (1, 68) = 107.4, *p* = 0.000), self-other overlap (*F* (1, 68) = 222.72, *p* = 0.000), and perceived similarity (*F* (1, 68) = 10.99, *p* = 0.001) before and after the virtual interaction (see Table 1). Taken together, the MANOVA results suggest that similarly to face-to-face interactions, virtual social interactions may induce pro-social effects.

**Table 1:** Descriptive statistics for composite average scores for each dyad on all questionnaires before and after interaction.

	Before the interaction		After the interaction	
	M	SD	M	SD
Positive affect*	32.39	5.64	35.8	5.08
Negative affect*	11.88	3.09	9.8	2.76
Trust*	14.57	1.95	15.67	2.17
Liking*	16.92	1.76	17.86	1.9
Cohesion*	130.2	48.36	160.8	50.84
Self-other overlap*	41.43	17.16	57.43	15.75
Perceived similarity*	55.87	7.99	58.06	8.77

*p < 0.01.

After validating that motor synchrony, emotional alignment and pro-social effects indeed arise during the virtual social interaction, a simple linear regression analysis was conducted to examine whether emotional alignment and the change in pro-social effects are associated with synchrony. To examine these associations, we first calculated the level of emotional alignment for both negative affect and positive affect using the difference in affect indexes. For both affects, we subtracted the index before the interaction from the index after the interaction. Next, we calculated the change that occurred in pro-social effects by subtracting the average dyad score after the interaction from the average dyad score before the interaction, this calculation was done separately for each of the pro-social effects.

In sum, the regression model included synchrony as the dependent variable and as independent variables were the emotional alignment indexes (negative and positive) and the changes in each of the pro-social effects (trust, liking, cohesion, self-other overlap, perceived similarity). The overall regression model was significant *R*^*2*^ = 0.293, *F* (7, 61) = 3.62, *p* = 0.003. Emotional alignment in positive affect (β = -−  0.394, *p* < 0.001) and the change in liking (β = 0.496, *p* < 0.001) were found to significantly predict the level of motor synchrony (see Table 2). Thus suggesting, that emotional alignment and pro-social effects were associated with the level of synchrony during the virtual interactions. In particular, a higher level of synchrony during the virtual interaction was found to be associated with increased positive emotional alignment and liking.

**Table 2:** Reggression coeffisionts of emotional alignment and pro-social effects on motor synchrony during virtual interactions.

	B	SE	β	t
Positive emotional alignment *	− 0.03	0.001	0.394	− 3.484
Negative emotional alignment	0.001	0.001	0.104	0.931
Trust	− 0.009	0.005	− 0.291	− 2.037
Liking*	0.018	0.005	0.496	3.650
Cohesion	0.000	0.000	− 0.093	− 0.643
Self-other overlap	0.000	0.001	0.50	-0.369
Perceived similarity	− 0.001	0.001	− 0.155	− 1.302

*p < 0.01.

## Discussion

Extensive research has been conducted on the correlation between motor synchrony and emotional alignment during face-to-face interactions^23,24,32,33^. Likewise, the connection between motor synchrony and pro-social effects has also been extensively documented^12–18^. However, it remains an open question whether these associations also exist in virtual social interactions. To address this question, the current study examined whether motor synchrony, emotional alignment, or other pro-social effects can be observed during virtual social interactions. Our findings reveal supporting evidence for the presence of both motor synchrony and emotional alignment during virtual social interactions. Moreover, we found that following the interaction participants reported a reduction in negative affect and an increase in positive affect, as well as greater feelings of liking, trust, perceived similarity, cohesiveness, and self-other overlap. Finally, our results suggest that higher level of synchrony is specifically linked to increased positive emotional alignment and liking. Thus, these results underscore the possibility for humans to communicate remotely, as this virtual social interaction seems to provide similar opportunity for connectedness as real-life social interactions.

In the current study, we instructed participants to share personal difficulties with one another during a virtual social interaction in order to generate a shared emotional experience. Our results indicate that while participants were instructed to share difficult experiences with one another, the interaction was accompanied by a reduction in negative affect and an upswing in positive affect. It is thus suggested that although unpleasant experiences were shared, the interaction itself was pleasant. A previous study has found that the mere acknowledgement that someone is sharing an emotional experience with us, without even communicating, makes the experience more pleasant^56^. Taken together, these results indicate the powerful effect of sharing emotional experiences with others, even when the interaction is virtual. Similarly, it has been suggested, that digital technology may assist in reducing loneliness and mitigate the negative effects of social distancing^57,58^. For example, recent studies have shown that increased virtual social interactions with close others during the pandemic were linked to higher levels of well-being in both younger and older adults^59,60^. Moreover, the Zoom platform allowed for forming and maintaining rapport between researcher and participant in a recent study^61^. Nonetheless, it is still unclear what causes this powerful effect of virtual social interactions.

To unravel this query and shed light on a question that is at the forefront of social psychologists today—that is, whether virtual social interactions provide the same opportunity for connectedness as real-life social interactions—we will take into consideration our first goal. As expected, our first goal (i.e., to demonstrate that emotional alignment and motor synchrony can arise spontaneously during virtual social interactions) received supporting evidence from our results. Given the important role motor synchrony and emotional alignment play in our social lives, having found these phenomena in virtual social interactions may assist in explaining the powerful effect of such interactions. Moreover, we found that similarly to face-to-face interaction, virtual social interactions that include both audio and video may provide similar pro-social effects^13,14,18,19,62–65^. Specifically, in our novel paradigm, the level of synchrony was particularly associated with liking and positive emotional alignment. Altogether, it seems that many social phenomena occurring in face-to-face interactions also occur in virtual social interactions. Similarly, a recent study showed that virtual interactions were not different from face-to-face interactions in terms of trust, liking, perceived similarity, and synchrony^41^. Therefore, we suggest that these phenomena may be the underlying mechanisms that explain the connection-promoting effect social virtual interactions have.

However, while our study found evidence for the possibility of virtual social interactions to promote connectedness, Towner et al.^66^ did not find this effect. It has previously been suggested that virtual social interactions that include audio create stronger social bonds compared to interactions including only text^67^. In accordance with this suggestion, the disparity between our study and Towner et al.'s^66^ study might be explained by the type of virtual social interactions used. Here, virtual social interactions were conducted via Zoom, therefore the interaction included both audio and video. In Towner et al.'s^66^ study, virtual social interactions were examined spontaneously, and participants reported using mostly messaging/texting as their primary mode of virtual social interaction. Thus, it could be presumed that different methods of virtual social interactions provide different opportunities for connectedness. This hypothesis lines up with our suggestion regarding the underlying mechanism of the connection-promoting effect of virtual social interactions. That is, interactions including audio and video might generate more social phenomena (motor synchrony, emotional alignment, etc.) than interactions including text exclusively. Therefore, it could be hypothesized that different types of virtual social interactions provide different opportunities for social phenomena to occur. As such, the connection-promoting effect a virtual social interaction will have depends on the social phenomena elicited during the interaction.

Nonetheless, some studies have failed to replicate the association between motor synchrony and pro-social effects during a virtual interaction between human and virtual agent, that is although they included both audio and video^20,21^. Moreover, although there were no significant differences found in facial expression mimicry, feelings of closeness, and trust between videoconferencing and in-person interactions, videoconferencing has been shown to reduce interactional engagement and hinder creative collaboration capacity^41^. It appears that the positive effects of virtual interactions cannot be solely attributed to the social phenomena that occur during the interaction. Many other factors come into play when evaluating the social effects of different types of interactions, including the setting, participants, and purpose.

Previous studies have suggested that people are more likely to synchronize movement with those they like, feel close to, trust, and perceive as similar to themselves^13,14,18,19,62–65^. Additionally, a link between synchrony and emotional alignment has been previously observed^23,24,32,33^. In line with this, we observed an increase in pro-social effects following synchronized virtual interaction as well as an emergence of emotional alignment. However, one may argue that a direct reported association between synchrony and changes in pro-social effects and emotional alignment should be evident in order to draw this conclusion. To address this concern, we conducted a regression analysis to examine whether emotional alignment and changes in pro-social effects are associated with synchrony. The results indicate a positive association between synchrony and alignment in positive affect and liking. Thus, a higher level of synchrony during virtual interaction is associated with increased positive social alignment and liking. We believe that this may not have been discovered had we altered even one aspect of the paradigm. It is safe to assume that each interaction's unique nature produces distinct social phenomena and outcomes. As we still have much to learn about various types of interactions and their outcomes, further studies are essential.

Several limitations of the current study should be noted. First, this study did not have any kind of a comparison group, thus we cannot determine the similarities and differences between virtual settings and face-to-face settings. Furthermore, our study design does not allow for establishing a causal relationship between motor synchrony, emotional alignment, and the pro-social effects of the interaction. In addition, the demographics of our sample present a limitation due to a lack of diversity in age, an unequal ratio between men and women, and the fact that all participants attend the same university in Israel. Another limitation of the current study was the narrow region we recorded for motor synchrony analysis. The video recordings included only the head and shoulder region and it is possible that examination of the entire body would have provided different results, although it was not relevant for this study since during the interaction participants only viewed this part of their interaction partner. Finally, a research assistant was present during the interaction. Although we attempted to minimize their impact by turning off their camera and microphone, it is uncertain whether this was effective. As having an experimenter present during a dyadic interaction is a common practice in face-to-face settings^68–70^, it would be valuable to investigate the impact of their presence in future studies involving video settings. Regardless of these limitations, the current study provides a basis for future studies investigating social interactions in a virtual environment.

In conclusion, the current study suggests that both synchrony and emotional alignment may arise spontaneously in the virtual world. To our knowledge, this is the first study that has found evidence for the spontaneous emergence of both synchrony and emotional alignment during a virtual interaction. Moreover, this interaction induced pro-social effects. It could also be assumed that this interaction was pleasant for the participants since it raised their affect. Finally, a higher level of synchrony was specifically linked to positive emotional alignment and liking. Here, we created a specific paradigm that elicited specific social phenomena and outcomes, some of which are similar to previous findings, and some seem to be unique to this paradigm. Thus, emphasizing that each interaction's unique nature produces distinct phenomena. Taken together, these findings may pave the way for developing new intervention protocols aiming at dealing with the consequences of social distancing. Using our findings, future research can try to design different types of virtual protocols and examine their therapeutic results as well as motor synchrony and emotional alignment during the interventions. Moreover, future studies may recreate our paradigm in different types of virtual social interactions and examine the different social phenomena each type of interaction elicits.

## Data Availability

The datasets generated and analyzed during the current study are available from the corresponding author upon reasonable request.
