# Author Correction: Virtual social interactions during the COVID-19 pandemic: the effect of interpersonal motor synchrony on social interactions in the virtual space

**DOI:** 10.1038/s41598-023-39048-y

**Published:** 2023-07-20

**Authors:** Hila Gvirts, Lya Ehrenfeld, Mini Sharma, Moran Mizrahi

**Affiliations:** Department of Psychology, Ariel University, 9851328 Ariel, Israel

Correction to: *Scientific Reports* https://doi.org/10.1038/s41598-023-37218-6, published online 28 June 2023

The original version of this Article contained an error in the name of Moran Mizrahi, which was incorrectly given as Moran Mizrachi.

The original Article has been corrected.

